# Loneliness and its association with depressed mood, anxiety symptoms, and sleep problems in Europe during the COVID-19 pandemic

**DOI:** 10.1017/neu.2020.48

**Published:** 2021-01-11

**Authors:** Ziggi Ivan Santini, Ai Koyanagi

**Affiliations:** 1 The Danish National Institute of Public Health, University of Southern Denmark, Studiestraede 6, 1455 Copenhagen, Denmark; 2 Parc Sanitari Sant Joan de Déu, CIBERSAM, Dr Antoni Pujadas, 42, 08830, Sant Boi de Llobregat, Barcelona, Spain; 3 ICREA, Pg. Lluis Companys 23, Barcelona, Spain

**Keywords:** COVID-19, depression, anxiety, sleep problems, loneliness

## Introduction

The ongoing COVID-19 pandemic has taken a toll on mental health across the globe. A number of reviews have suggested that the pandemic may have had an overwhelmingly negative influence on mental health worldwide (Xiong *et al*., 2020; Bueno-Notivol *et al*., 2021). For example, a meta-analysis conducted by Bueno-Notivol *et al*. (2021) suggests a 7-fold increase in depression prevalence across six countries (three European and three Asian countries) as compared to a global depression prevalence in 2017. In 2017, The World Health Organisation’s Global Burden of Disease Study has identified depression as being the single largest contributor to global disability across all diseases, and anxiety ranking sixth (WHO, 2017). In other words, mental health problems were a pressing global concern well before the discovery of COVID-19. However, as a result of the COVID-19 pandemic, global disability due to mental health problems is likely to escalate even further without intervention and appropriate health and social policies in place (Holmes *et al*., 2020). Additionally, while the virus itself has posed a significant threat to the lives of older adults in particular, it is not the only concern that affects this age group. Depression and anxiety in older adulthood are major public health issues due to their high prevalence and poor outcomes, such as impairment in various types of functioning, self-neglect, and ultimately a risk for premature mortality and suicide (Fiske *et al*., 2009; Wolitzky-Taylor *et al*., 2010). It is predicted that there will be an upsurge in mental health service demand as such adverse outcomes may increase (Holmes *et al*., 2020). The consequences of compromised mental health due to the COVID-19 pandemic may – apart from the suffering of those directly affected – also severely impede health and social care systems on a global scale.

The pervasive and abrupt changes that have occurred as a result of the pandemic are likely to have had a profound effect on social well-being, which in turn may have influenced mental health (Chen *et al*., 2020; Manca *et al*., 2020). A long line of research has documented an increased risk of mental health problems associated with loneliness (Santini *et al*., 2020). Loneliness is likely to have accumulated during the pandemic due to mobility restrictions, quarantines, social distancing, and the like. Although some studies have reported associations between loneliness and mental health problems in single countries during the pandemic, there is a need to explore such associations in populations across the entire European continent. Therefore, the aim of the present study was to assess: (a) the risk of mental health problems (depressed mood, anxiety symptoms, sleep problems) associated with loneliness during the COVID-19 pandemic; (b) the risk of mental health problems associated with worsened loneliness since the COVID-19 outbreak; (c) the risk of worsened mental health problems since the COVID-19 outbreak associated with loneliness; and (d) the risk of worsened mental health problems since the COVID-19 outbreak associated with worsened loneliness since the COVID-19 outbreak.

## Methods

Data stem from the Survey of Health, Ageing, and Retirement in Europe (SHARE) COVID-19 survey (Börsch-Supan, 2020). SHARE is a European bi-annual, cross-national, and longitudinal research project collecting nationally-representative data among community-dwelling participants aged 50 years and above at the time of data collection (2020). Participants in 26 European countries (see Appendix 1 in Supplementary material) were contacted and interviewed via telephone (computer assisted telephone interview – CATI) in the period June-August 2020 (the survey also included Israel, but we did not include it in the study because it is not part of Europe, neither did we include household members of the respondent that were less than 50 years old). A total of 50 609 participants were included in this cross-sectional study.

We included six outcomes pertaining to self-reported symptoms, all assessed using single-item questions: (a) any depressed mood; (b) worsened depressed mood; (c) any anxiety symptoms; (d) worsened anxiety symptoms; (e) any sleep problems; (f) worsened sleep problems. To assess any depressed mood, any anxiety symptoms, and any sleep problems, the respondents were asked ‘In the last month, have you been sad or depressed?’, ‘In the last month, have you felt nervous, anxious, or on edge?’, and ‘Have you had trouble sleeping recently?’, respectively, with yes or no answer options. For those who answered affirmatively to these questions, they were further asked ‘Has that been more so, less so, or about the same as before the outbreak of Corona?’ Any depressed mood, any anxiety symptoms, and any sleep problems were operationalised as present (yes) or absent (no). Worsened depressed mood, anxiety symptoms, or sleep problems were operationalised as present (more so) or absent (less so or about the same).

Our predictor was loneliness and worsened loneliness. For loneliness, the respondent was asked ‘How much of the time do you feel lonely?’, with response options being often, some of the time, or hardly ever or never. Worsened loneliness was assessed among those responding often or some of the time. These respondents were asked ‘Has that been more so, less so, or about the same as before the outbreak of Corona?’. Loneliness was operationalised as present (often or some of the time) or absent (hardly ever or never – reference category). Worsened loneliness was operationalised as present (more so) or absent (less so or about the same – reference category).

Multivariable logistic regression analyses were conducted to assess the associations between loneliness/worsened loneliness and all six outcomes, while adjusting for country, gender, age, marital/partnership status, employment status, income, financial strain, self-rated health, and anyone close to the respondent having died due to COVID-19. See Appendix 1 in Supplementary material for more information about included countries, data, methodology, covariates, missing data, and sample characteristics. All variables were included in the model as categorical variables with the exception of age (continuous variable). In all analyses, weights to adjust for different selection probabilities, sampling error, non-response bias, and population size were taken into account to generate nationally-representative estimates using the Stata svy command. Conventionally, *p*-values < 0.05 were considered statistically significant.

## Results

The prevalence of depressed mood, anxiety symptoms, and sleep problems were 28.6%, 30.4%, and 27.3%, respectively, with the prevalence of worsening among these conditions being 63.5%, 73%, and 34.6%, respectively (see Appendix 1). The prevalence of loneliness was 29.4% and among those reporting loneliness, the prevalence of worsened loneliness was 39.9%. The results from the multivariable logistic regressions are shown in Table 1. Loneliness and worsened loneliness were both associated with significantly higher odds for any depressed mood, any anxiety symptoms, and any sleep problems (OR = 1.47, 4.37). Loneliness was significantly associated with worsened anxiety symptoms and sleep problems but not worsened depressed mood. Worsened loneliness was significantly associated with particularly strong risk for worsened depressed mood (OR = 10.11; 95% CI = 8.06, 12.7), anxiety symptoms (OR = 7.68; 95% CI = 6.10, 9.65), and sleep problems (OR = 6.26; 95% CI = 4.78, 8.20).

In terms of death among close social ties due to COVID-19, we also tested for effect modification in all the models (i.e. if death among close social ties due to COVID-19 moderated the associations), but we did not find any significant interactions (results not shown).

**Table 1.** Associations of loneliness or worsened loneliness since COVID-19 outbreak (independent variables) with any mental health problem or worsened mental health problem since outbreak (dependent variables) among older adults (aged 50+ years old) in Europe (interviewed June–August 2020) estimated by multivariable logistic regression

	OR	95% CI	*p*-value	OR	95% CI	*p*-value
	Any depressed mood			Worsened depressed mood		
Loneliness						
Absent	1			1		
Present	4.37	3.91, 4.89	<0.001	0.99^a^	0.83, 1.18	0.890
Worsened loneliness						
Absent	1			1		
Present	2.23^b^	1.90, 2.62	<0.001	10.11^c^	8.06, 12.7	<0.001

	Any anxiety symptoms			Worsened anxiety symptoms		
Loneliness						
Absent	1			1		
Present	2.70	2.43, 3.00	<0.001	1.33^d^	1.11, 1.59	0.002
Worsened loneliness						
Absent	1			1		
Present	2.21^b^	1.88, 2.59	<0.001	7.68^e^	6.10, 9.65	<0.001

	Any sleep problems			Worsened sleep problems		
Loneliness						
Absent	1			1		
Present	2.08	1.86, 2.32	<0.001	1.79^f^	1.47, 2.18	<0.001
Worsened loneliness						
Absent	1			1		
Present	1.47^b^	1.25, 1.72	<0.001	6.26^g^	4.78, 8.20	<0.001

OR, odds ratio; CI, confidence interval.

All models adjusted for country, gender, age, marital/partnership status, employment status, income, financial strain, self-rated health, and anyone close to the respondent having died due to Covid-19.

a The sample was restricted to individuals reporting any depressed mood (*N* = 13 099).

b The sample was restricted to individuals reporting loneliness (*N* = 14 393).

c The sample was restricted to individuals reporting loneliness and any depressed mood (*N* = 7272).

d The sample was restricted to individuals reporting any anxiety symptoms (*N* = 15 175).

e The sample was restricted to individuals reporting loneliness and any anxiety symptoms (*N* = 6959).

f The sample was restricted to individuals reporting any sleep problems (*N* = 13 966).

g The sample was restricted to individuals reporting loneliness and any sleep problems (*N* = 6013).

## Discussion

According to our data, about 30% of the European population (aged 50+ years) report experiencing a mental health problem (i.e. depressed mood, anxiety symptoms, or sleep problems) during the COVID-19 pandemic. Moreover, about two thirds (64%–73%) of those experiencing depressed mood or anxiety symptoms report that these conditions have worsened since the outbreak of the pandemic. The corresponding figure for worsened sleep problems was 35%. These results suggest that the pandemic has led to deteriorating mental health in the European population of older adults. Similarly, our results suggest that the pandemic may have brought about considerable declines in social well-being. Among participants reporting loneliness, about 40% felt more lonely since the outbreak than previously. Most importantly, our results reveal significant associations between loneliness and mental health problems during the pandemic. Having adjusted for COVID-19 mortality, our results suggest that these associations are not accounted for by people having lost one or more close family members, confidants, friends or other kinds of close relationships due to COVID-19 infection. In other words, the patterns in our results appear to be features of the pandemic in general rather than a consequence of COVID-19 mortality.

Before going into the results further, it is necessary to keep the following limitations in mind when interpreting them: First, interviews were conducted in the period June-August 2020, when infection numbers across Europe were relatively low and there were relatively fewer government restrictions than previously and later on. Most countries have seen increases in infection rates at the beginning of the winter season 2020, and it is possible that performing the interviews during this time would produce different and possibly even stronger and more alarming results. Thus, the results we present may be regarded as conservative estimates. Second, data were self-reported, and there is a risk of self-report bias and issues pertaining to common-methods variance. Third, we used single-item questions to assess symptoms of mental health problems and loneliness rather than validated scales or medical diagnoses (which were not included in the SHARE COVID-19 survey). Finally, the cross-sectional design precludes us from making causal inferences. The use of retrospective variables (i.e. ‘Has that been more so, less so, or about the same as before the outbreak of Corona?’) may have partly compensated for this, as they inquire about change over time. It is also important to note that the relationship between loneliness and mental health problems is likely bi-directional (Santini *et al.*, 2020). In other words, these factors may be reinforcing each other over time in a downward spiral unless buffered by protective factors or interrupted through intervention.

While a multitude of factors may have affected population mental health, such as the virus itself as well as other consequences of the pandemic (e.g. economic recession, financial insecurity, heavy exposure of the pandemic in the media), the social consequences of the pandemic cannot be disregarded. The risk of infection, social distancing measures, government restrictions and quarantines have resulted in unfavorable social conditions that in turn may have had negative implications for mental health. In line with recent research (Manca *et al.*, 2020), the current study shows strong links between loneliness and mental health problems during the pandemic. This is particularly so among those that reported any amount of both loneliness *and* mental health problems (about 12%–16%). Among such individuals, worsened loneliness during the pandemic was associated with an extremely high risk for worsened depressed mood, anxiety symptoms, and sleep problems (OR = 6.26, 10.11). In conclusion, our data suggest an escalation in loneliness across Europe since the outbreak of the pandemic, which is associated with a dramatic increase in odds for deteriorating mental health problems. There is an urgent need for research to address the mental health implications of intensified loneliness experienced during the pandemic, as well as how loneliness and the consequences of it may be mitigated under pandemic conditions. The social ramifications of the pandemic must also be considered and prioritised accordingly in health policy, planning and prevention strategies.

## Supplementary material

For supplementary material accompanying this paper visit https://doi.org/10.1017/neu.2020.48. click here to view supplementary material
