# Supplementary material for: Loneliness and its association with depressed mood, anxiety symptoms, and sleep problems in Europe during the COVID-19 pandemic
Source: Acta Neuropsychiatr. 2021 Jan 11:1–4. doi: 10.1017/neu.2020.48 (PMC7844146; doi:10.1017/neu.2020.48)
Supplement: Supplementary file 1 [file S0924270820000484sup.zip › S0924270820000484sup002.docx]

Appendix 1

Table A1 shows the included countries and the number of participants in each country. Table A2 shows the characteristics of the study sample and the various covariates and categories. The covariates included gender, age, marital/partnership status, current employment status, income since outbreak in tertiles, financial strain (ability to make ends meet since outbreak), and self-rated health. We created a ‘missing’ category for income tertiles, because 24.8% was missing. All statistical models were based on the sample with no missing data (complete case analysis), and the proportion of missing data within the study sample (N=50,609) were as follows: sex 0.0%; age 0.0%; marital/partnership status 0.0%; employment status 0.6%; income 0.0% (24.8% without ‘missing’ category); financial strain 3.1%; any depressed mood 0.8%; any anxiety symptoms 0.8%; any sleep problems 0.7%; loneliness 1.0%; anyone close to the respondent having died to COVID-19 0.5%. For the subsamples (those reporting any depressed mood N=13,099; anxiety symptoms N=15,175, sleep problems N=13,966, loneliness N=14,393) missing data were: Worsened depressed mood 0.2%; worsened anxiety symptoms 0.1%; worsened sleep problems 0.1%; worsened loneliness 0.1%, respectively.

| **Table A1.** Countries and participants. | |
| --- | --- |
| Country | Participants (N) |
| Germany | 2,649 |
| Sweden | 1,364 |
| The Netherlands | 787 |
| Spain | 2,052 |
| Italy | 3,696 |
| France | 2,052 |
| Denmark | 1,996 |
| Greece | 3,614 |
| Switzerland | 1,880 |
| Belgium | 3,777 |
| Czech Republic | 2,629 |
| Poland | 2,920 |
| Luxembourg | 928 |
| Hungary | 1,000 |
| Portugal | 1,114 |
| Slovenia | 3,107 |
| Estonia | 4,519 |
| Croatia | 2,001 |
| Lithuania | 1,260 |
| Bulgaria | 813 |
| Cyprus | 796 |
| Finland | 1,460 |
| Latvia | 964 |
| Malta | 826 |
| Romania | 1,471 |
| Slovakia | 934 |
| **Total** | **50,609** |

| **Table A2.** Characteristics of the study sample. | | | |
| --- | --- | --- | --- |
| **Characteristic** | **Category** | **N** | **Weighted %** |
| **Total sample** |  |  |  |
| Total participants |  | 50,609 |  |
| Sex | Female | 29,205 | 54.0 |
| Age (years) | Mean (SD) | 70.5 (9.3) |  |
| Marital/partnership status | Married/with partner | 34,962 | 64,3 |
| Employment status | Retired or unemployed before outbreak and currently | 39,736 | 67.1 |
|  | Employed or self-employed before outbreak and currently | 8,669 | 26.2 |
|  | Lost employment due to outbreak | 1,908 | 6.7 |
| Income since outbreak in tertiles | Low | 12,740 | 18.3 |
|  | Middle | 12,927 | 23.4 |
|  | High | 12,546 | 33.5 |
|  | Missing | 12,396 | 24.8 |
| Ability to make ends meet since outbreak | With great difficulty | 4,583 | 8.3 |
|  | With some difficulty | 12,912 | 23.8 |
|  | Fairly easy | 17,120 | 36.0 |
|  | Easy | 14,432 | 31.8 |
| Self-rated health | Excellent | 3,336 | 7.2 |
|  | Very good | 7,920 | 17.8 |
|  | Good | 22,348 | 47.0 |
|  | Fair | 13,130 | 22.1 |
|  | Poor | 3,602 | 5.9 |
| Anyone close died due to Covid-19 | Yes | 1,335 | 3.3 |
| Any depressed mood | Present | 13,099 | 28.6 |
| Any anxiety symptoms | Present | 15,175 | 30.4 |
| Any sleep problems | Present | 13,966 | 27.3 |
| Loneliness | Present | 14,393 | 29.4 |
| **Subsamples** |  |  |  |
| Worsened depressed mood^a^ | Present | 8,255 | 63.5 |
| Worsened anxiety symptoms^b^ | Present | 10,759 | 73.0 |
| Worsened sleep problems^c^ | Present | 4,036 | 34.6 |
| Worsened loneliness^d^ | Present | 5,782 | 39.9 |
| Sampling weights were used for the calculation of proportions and means (SD).  ^a^ The sample was restricted to individuals reporting any depressed mood (N=13,099)  ^b^ The sample was restricted to individuals reporting any anxiety symptoms (N=15,175)  ^c^ The sample was restricted to individuals reporting any sleep problems (N=13,966)  ^d^ The sample was restricted to individuals reporting loneliness (N=14,393) | | | |
