# Supplementary material for: Loneliness and its association with depressed mood, anxiety symptoms, and sleep problems in Europe during the COVID-19 pandemic
Source: Acta Neuropsychiatr. 2021 Jan 11:1–4. doi: 10.1017/neu.2020.48 (PMC7844146; doi:10.1017/neu.2020.48)
Supplement: Supplementary file 1 [file S0924270820000484sup.zip › S0924270820000484sup001.pdf]

Appendix 1

Table A1 shows the included countries and the number of participants in each country. Table A2 shows the characteristics of the study sample and the various covariates and categories. The covariates included gender, age, marital/partnership status, current employment status, income since outbreak in tertiles, financial strain (ability to make ends meet since outbreak), and self-rated health. We created a 'missing' category for income tertiles, because 24.8% was missing. All statistical models were based on the sample with no missing data (complete case analysis), and the proportion of missing data within the study sample (N=50,609) were as follows: sex 0.0%; age 0.0%; marital/partnership status 0.0%; employment status 0.6%; income 0.0% (24.8% without 'missing' category); financial strain 3.1%; any depressed mood 0.8%; any anxiety symptoms 0.8%; any sleep problems 0.7%; loneliness 1.0%; anyone close to the respondent having died to COVID-19 0.5%. For the subsamples (those reporting any depressed mood N=13,099; anxiety symptoms N=15,175, sleep problems N=13,966, loneliness N=14,393) missing data were: Worsened depressed mood 0.2%; worsened anxiety symptoms 0.1%; worsened sleep problems 0.1%; worsened loneliness 0.1%, respectively.

Table A1. Countries and participants.

Country	Participants (N)
Germany	2,649
Sweden	1,364
The Netherlands	787
Spain	2,052
Italy	3,696
France	2,052
Denmark	1,996
Greece	3,614
Switzerland	1,880
Belgium	3,777
Czech Republic	2,629
Poland	2,920
Luxembourg	928
Hungary	1,000
Portugal	1,114
Slovenia	3,107
Estonia	4,519
Croatia	2,001
Lithuania	1,260
Bulgaria	813
Cyprus	796
Finland	1,460
Latvia	964
Malta	826
Romania	1,471
Slovakia	934
**Total**	**50,609**

Table A2. Characteristics of the study sample.

Characteristic	Category	N	Weighted %
**Total sample**			
Total participants		50,609	
Sex	Female	29,205	54.0
Age (years)	Mean (SD)	70.5 (9.3)	
Marital/partnership status	Married/with partner	34,962	64,3
Employment status	Retired or unemployed before outbreak and currently	39,736	67.1
	Employed or self-employed before outbreak and currently	8,669	26.2
	Lost employment due to outbreak	1,908	6.7
Income since outbreak in tertiles	Low	12,740	18.3
	Middle	12,927	23.4
	High	12,546	33.5
	Missing	12,396	24.8
Ability to make ends meet since outbreak	With great difficulty	4,583	8.3
	With some difficulty	12,912	23.8
	Fairly easy	17,120	36.0
	Easy	14,432	31.8
Self-rated health	Excellent	3,336	7.2
	Very good	7,920	17.8
	Good	22,348	47.0
	Fair	13,130	22.1
	Poor	3,602	5.9
Anyone close died due to Covid-19	Yes	1,335	3.3
Any depressed mood	Present	13,099	28.6
Any anxiety symptoms	Present	15,175	30.4
Any sleep problems	Present	13,966	27.3
Loneliness	Present	14,393	29.4
**Subsamples**			
Worsened depressed mood[a]	Present	8,255	63.5
Worsened anxiety symptoms[b]	Present	10,759	73.0
Worsened sleep problems[c]	Present	4,036	34.6
Worsened loneliness[d]	Present	5,782	39.9

Sampling weights were used for the calculation of proportions and means (SD).

[a] The sample was restricted to individuals reporting any depressed mood (N=13,099)

[b] The sample was restricted to individuals reporting any anxiety symptoms (N=15,175)

[c] The sample was restricted to individuals reporting any sleep problems (N=13,966)

[d] The sample was restricted to individuals reporting loneliness (N=14,393)
